# Characterization of the Second Harmonic Generation of Boron Nitride Nanotube Macroscopic Assemblies

**DOI:** 10.3390/nano15110861

**Published:** 2025-06-03

**Authors:** Ping Lu, Jingwen Guan, Cyril Hnatovsky, Huimin Ding, Kasthuri De Silva, Liliana Gaburici, Christopher Kingston, Stephen J. Mihailov

**Affiliations:** Quantum and Nanotechnologies Research Center, National Research Council Canada, 100 Sussex Drive, Ottawa, ON K1A 0R6, Canada; jingwen.guan@nrc-cnrc.gc.ca (J.G.); kyrylo.hnatovsky@nrc-cnrc.gc.ca (C.H.); huimin.ding@nrc-cnrc.gc.ca (H.D.); manjula.desilva@nrc-cnrc.gc.ca (K.D.S.); liliana.gaburici@nrc-cnrc.gc.ca (L.G.); christopher.kingston@nrc-cnrc.gc.ca (C.K.); stephen.mihailov@nrc-cnrc.gc.ca (S.J.M.)

**Keywords:** boron nitride nanotubes (BNNTs), second harmonic generation (SHG), femtosecond infrared (fs-IR) laser, nonlinear optics

## Abstract

Boron nitride nanotubes (BNNTs) are predicted to be promising one-dimensional nonlinear optical materials, but to date, only one experimental observation has been made using individual nanotubes. In this work, second harmonic generation (SHG) was achieved from free-standing bulk BNNT sheets and BNNT coatings on silica substrates. Focusing femtosecond infrared (fs-IR) laser pulses with a wavelength of 800 nm onto the BNNT assemblies resulted in strong SHG at a wavelength of 400 nm. It was observed that due to the thickness variation of the BNNT assemblies and orientational alignment of BNNTs in the assemblies, the intensity of the second-harmonic (SH) radiation changed dramatically when different locations on the samples were investigated. Among all the BNNT assemblies tested, the localized SH response and its dependence on the polarization of the excitation fs-IR pulses were the strongest in BNNT coatings produced by a dip-coating process. By measuring the SH response, the uniformity, reproducibility, and efficiency of BNNT deposition processes could be assessed. For applications requiring a high SH response from BNNT assemblies, the process of dip coating is preferred.

## 1. Introduction

Conceptually, a single-walled boron nitride nanotube (BNNT) is formed when a single sheet of hexagonal boron nitride (h-BN) rolls onto itself, whereas a multi-walled BNNT is formed when multiple sheets of h-BN roll onto themselves. Similar to carbon nanotubes (CNTs), in which a hexagonal network of carbon atoms constructs a one-dimensional tubular nano-structure with a high length-to-diameter ratio, the forming hexagonal network of BNNTs is made of alternating boron and nitrogen atoms. However, unlike the case of CNTs, the synthesis of BNNTs is quite challenging. Since BNNTs were first synthesized through arc discharge in 1995 [1], developing an efficient BNNT production route has been a significant issue because of the low yield of the proposed synthesis methods and the poor quality of the resultant BNNTs. Fortunately, many great successes in BNNT synthesis have been achieved in recent years, enabling access to this new emerging nanomaterial and paving the way for the development of promising applications. These synthesis methods utilize laser ablation, chemical vapor deposition, ball milling, pyrolysis, arc-jet plasma and inductively thermal-coupled plasma (ICP). In particular, the ICP technology has been extensively used for the synthesis of a wide range of nano-structured materials [2,3,4,5,6,7,8,9,10] and has received considerable attention for its promise to produce large volumes of high-quality BNNTs [11,12].

Owing to their unique atomic structure and extraordinary characteristics, BNNTs have numerous excellent intrinsic properties, such as superior mechanical strength, light weight, high thermal conductivity, high thermal stability, high oxidation resistance, and strong neutron-shielding capability. Unlike CNTs, BNNTs have a wide band gap (~5.5 eV) because of the polarization in B-N bonds caused by the difference in electronegativity of boron (2.04) and nitrogen (3.04) [13,14], which makes them electrically insulating and transparent in the visible spectral region [15]. The bandgap of BNNTs is not dependent on the diameters and chirality of the nanotubes [16]; however, they are sensitive to doping [17,18,19,20] and functionalization [21,22]. These properties of BNNTs have triggered great interest in fundamental studies on using BNNTs in mechanical reinforcement nanocomposites, transparent bulk composites, strong lightweight hybrid materials, and high-temperature-resistant materials, as well as applications of these emerging materials in various harsh environments featuring high temperatures, strong electromagnetic fields, high corrosion, and high-dose ionizing radiation [23,24,25,26,27]. Due to its highly polarized B-N bonds [28], the geometrical structure of h-BN is not symmetric, which is necessary for the occurrence of second-order nonlinear processes. The second harmonic (SH) responses of monolayer and multilayer h-BN flakes have been experimentally studied [29,30,31] and strong SH responses were observed in h-BN sheets with an odd number of layers. For BNNTs, theoretical calculations found that both zigzag and chiral BNNTs exhibit a nonlinear susceptibility that is much larger than that of bulk h-BN materials [32]. Another theoretical work on the second-order nonlinear response of arrays of uniformly sized and well-aligned zigzag single-walled BNNTs of fairly large diameters showed the promise of BNNTs for various electro-optical applications [33]. However, there is still a lack of experimental studies on the nonlinear optical responses and structure-dependent behavior of the nonlinear optical responses of BNNTs. The first report on SHG in a large diameter BNNT was published in 2024, where it was shown that the efficiency of SHG depends on its length, diameter, number of walls, and polarity [34]. Usually, SHG in multiwalled BNNTs is weak due to the diverse chirality, handedness, and polarity of each individual BNNT. However, in some cases individual multi-walled BNNTs exhibit a preference for coherently stacked configurations and, as a result, the ability to generate a strong SH signal. Such an effect has many potential applications in encrypted communication and quantum information [34].

In this work, to the best of our knowledge, the SH responses of BNNT macroscopic assemblies are reported for the first time. BNNT coatings on silica substrates and as free-standing BNNT sheets were exposed to a femtosecond infrared (fs-IR) laser beam focused onto the corresponding BNNT assemblies. Large variations in the SH responses were observed when different locations on the BNNT macroscopic assemblies were probed. The dependence of SHG on the polarization of the fs-IR laser pulse was also investigated. Different BNNT assemblies with maximized/minimized SHG were experimentally examined and it was shown that the monitoring of SHG in BNNT assemblies can be used as an effective way to characterize their uniformity. In particular, the polarization dependence of the SH response can be used for evaluating the BNNT alignment in the macroscopic assemblies. Our analysis also showed that BNNT coatings produced by the dip-coating process exhibit the strongest SH response.

## 2. Experimental Results and Discussion

### 2.1. Experiment Setup

**Figure 1:**
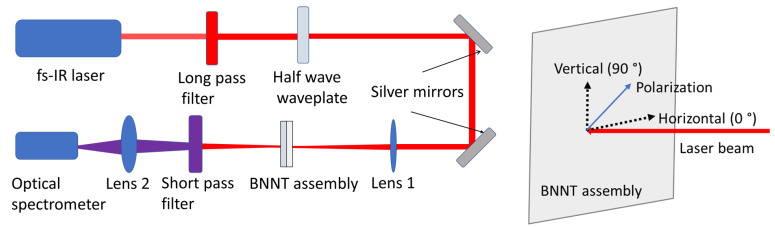
Optical setup to generate and measure the SH signal from the BNNT macroscopic assembly (**left**); polarization of the laser beam (**right**).

SHG in two types of BNNT macroscopic assemblies, free-standing BNNT sheets and BNNT coatings on silica substates, was studied in this work. The experimental setup to generate and collect the SH signal is schematically shown in Figure 1. A femtosecond infrared (fs-IR) laser beam was focused onto the BNNT assembly and the SH signals generated in the sample were collected using a spectrophotometer. The fs-IR beam with a central wavelength of 800 nm was from a Ti/sapphire regenerative amplifier (Spitfire, Spectra-Physics). The repetition rate of the laser was 1 KHz and the pulse duration was approximately 100 fs. It was found in the experiments that, along with the high-intensity fs-IR laser wavelength, a weak signal centered around 400 nm was also present. An optical long-pass filter (Edmund Optics, #62-982) was used to block all the wavelengths below 450 nm so that only the 800 nm wavelength of the laser beam was allowed to be focused on the BNNT assembly. The focal length of the lens that was used to focus the laser beam onto the BNNT assembly was 50 mm (lens 1 in Figure 1). Considering that the diameter of the fs-IR laser beam is approximately 11 mm, the calculated 1/e^2^ beam diameter is approximately 4.5 µm at the focal spot on the BNNT assembly. Because the SH intensity has a quadratic dependence on the incident laser intensity, the 1/e^2^ diameter of the SH signal generated in the BNNT assembly is approximately 3.2 µm. An optical short-pass filter was placed after the BNNT assembly to block the incident laser beam that was passing through the sample. Another lens (lens 2 in Figure 1) was then used to collect the signal originating from the BNNT assemblies and focus it at the entrance slit of an optical spectrophotometer (Flame, Ocean Optics). In order to assess the dependence of the SH signal on the polarization of the excitation laser beam, a half-wave plate was used to vary the orientation of the linear polarization of the incident laser beam on the BNNT assembly, as shown on the right side of Figure 1.

**Figure 2:**
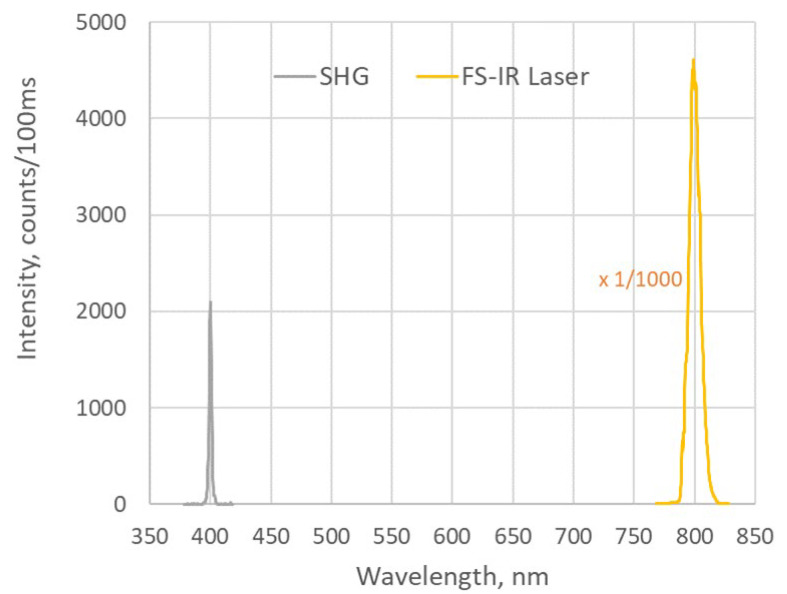
Spectra of the SH signal at a wavelength of 400 nm from a 35 µm thick BNNT sheet and the excitation light from the fs-IR laser at a wavelength of 800 nm.

**Figure 3:**
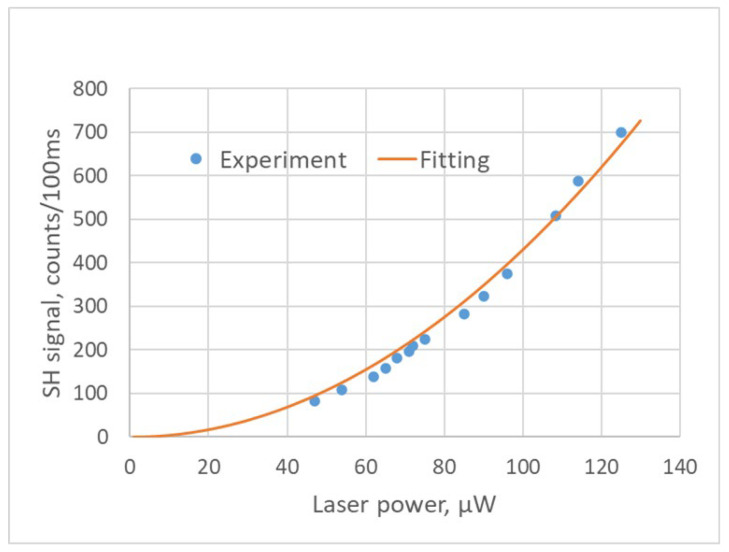
The dependence of the generated SH signal on the power of the excitation laser.

Figure 2 shows a typical spectrum of the signal produced in a 35 µm thick BNNT sheet along with the spectrum of the excitation signal at 800 nm from the fs-IR laser. It can be seen that the central wavelength of the signal from the BNNT sheet is exactly one half of the excitation wavelength, thus confirming that the collected signal at 400 nm is indeed from the SHG process. The SH signals at various excitation laser powers were measured as well; see the results in Figure 3. The equation of the fitting curve used in Figure 3 is $y=ax^{2}$, where a=0.043. It is clearly shown in Figure 3 that the SH signal has a quadratic dependence on the excitation laser power, confirming that the light signal at 400 nm collected by the optical spectrometer is indeed from the SHG process. It was observed in the experiment that the SH signal fluctuated significantly when the laser power was above 200 µW. This is likely due to the heating effect produced by the excitation laser light. When the laser power was under 160 µW, the fluctuation of SH signal was less than 5%. This remaining fluctuation could be due to the laser focus drift or the laser pulse energy fluctuation (3%). The experimental data presented in this work are the averaged values based on more than 10 SH signal measurements. The laser powers used in the experiments were 160 µW and below.

### 2.2. SHG in Free-Standing BNNT Sheets

**Figure 4:**
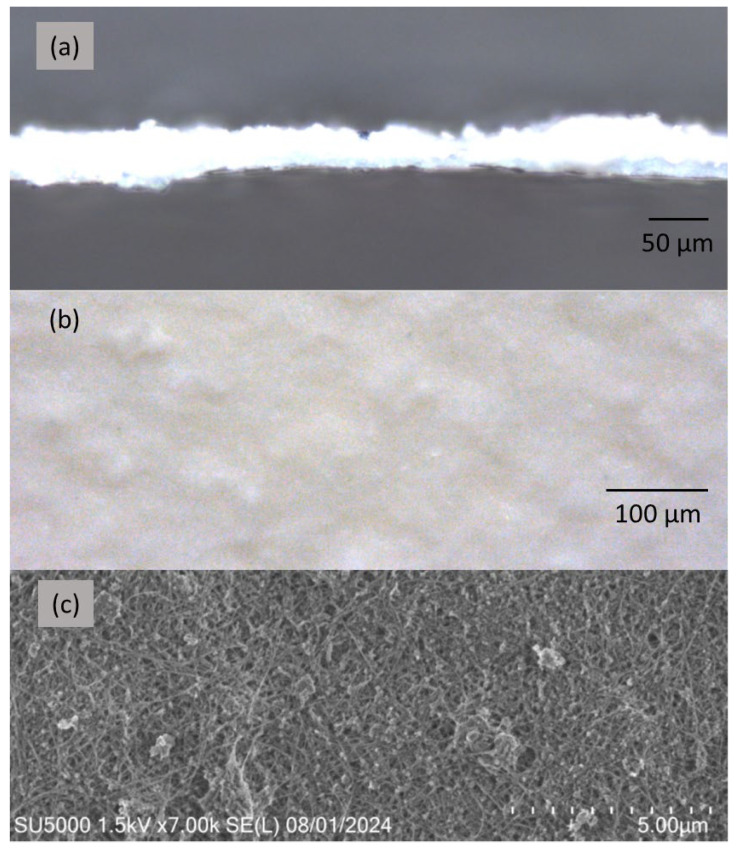
An optical microscopy image (**a**) of the cross-section of a BNNT sheet with an average thickness of 35 µm. Optical microscopy (**b**) and SEM (**c**) images of the surface of the BNNT sheet in (**a**).

In this work, three BNNT sheets with an average thickness of 18, 35, and 60 µm were tested by using the setup shown in Figure 1. The free-standing BNNT sheets were produced by a vacuum filtration method from a BNNT suspension in water using a polycarbonate filter membrane. The thicknesses of the BNNT sheets produced by this process were not the same at different locations on the sheets. An optical microscopy image of the cross-section of a BNNT sheet is shown in Figure 4a; the thickness of the sheet varied from 26 to 52 µm depending on the location, and the average thickness was around 35 µm. These thickness variations may be a factor affecting the SH signal intensity. Figure 4b and Figure 4c show an optical microscopy image and a scanning electron microscopy image (SEM), respectively, of the surface of the 35 µm thick BNNT sheet. As we can see from Figure 4c, the BNNT sheet is composed of individual BNNTs and BNNT bundles with empty spaces between them. The individual BNNTs and BNNT bundles are mainly randomly orientated on a large scale, i.e., they are not aligned along any particular direction on the surface. The optical microscopy and SEM images of the 18 and 60 µm thick BNNT sheets show similar morphologies.

**Figure 5:**
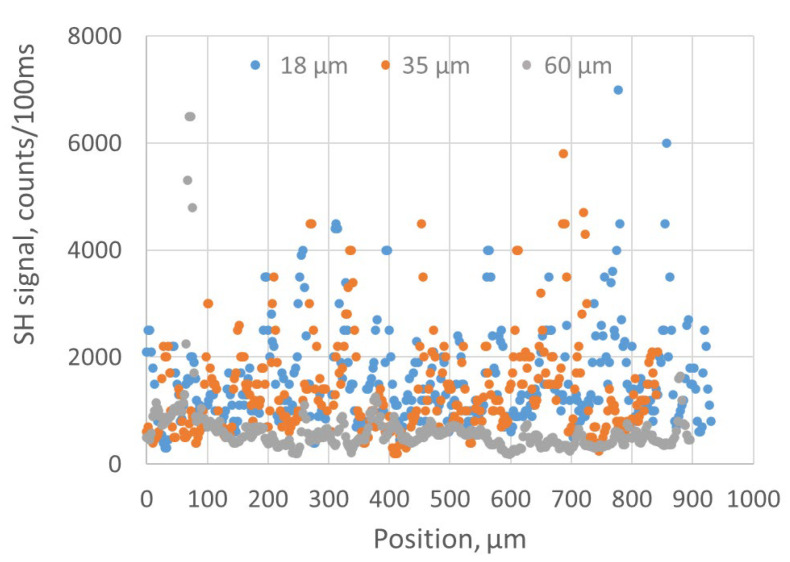
SH responses of the 18, 35, and 60 µm thick BNNT sheets at different locations on the samples.

The SH response of the BNNT sheet had large fluctuations when the fs-IR laser beam was focused at different locations of the BNNT sheet’s surface. Figure 5 shows the intensities of the SH signals generated in 18, 35, and 60 µm thick BNNT sheets at different locations. To obtain these data, the focus of the fs-IR laser beam formed by lens 1 was scanned along the surfaces of the BNNT sheets and the magnitudes of the respective SH signals were recorded with the optical spectrophotometer every time when the focus was displaced by 2.5 µm. The incident power of the fs-IR laser at each location was consistently 160 µW. As we can see, the SH response of the 60 µm thick BNNT sheet was much weaker than those of the 18 and 35 µm thick BNNT sheets. The averaged magnitudes of the SH signals were 1562, 1366, and 621 counts/100 ms for the 18, 35, and 60 µm coating thickness, respectively. The reduced SH response of the 60 µm thick BNNT sheet was likely caused by strong scattering of the generated SH signal at the numerous BNNT boundaries present in the thicker layer. As we can see from Figure 4c, the BNNT assembly is composed of BNNTs and air voids. The air void percentage in the BNNT assembly can be determined by measuring the effective refractive index of the assembly with a tapered fiber sensor [35], and can be as high as 50%. When the generated SH signal passes through the BNNT assembly with such a large percentage of air voids, high scattering loss is expected for thick BNNT assemblies. The maximum SHG efficiency of the sheets according to the spectrophotometric measurements, that is, the magnitude of the SH signal at 400 nm divided by the magnitude of the fs-IR excitation signal at 800 nm, was in the order of 10^−4^. Measuring the SH responses of a BNNT assembly at different locations has the potential to facilitate evaluating the thickness uniformity of the BNNT assembly; however, other parameters that may affect the measurement should also be considered. For example, the SH response may be affected by the local inhomogeneities of the BNNT assembly, such as local volume density/air void percentage, bundle sizes and randomness of BNNTs, etc. The focused laser beam geometry is another factor that may affect the SH response of thick BNNT assemblies. As shown in Figure 1, the laser beam was focused onto the BNNT assembly by using a lens with a focal length of 50 mm (lens 1). Based on the diameter and wavelength of the incident beam, the calculated depth of field of the lens (i.e., twice the Rayleigh range) is around 40 µm. The variation in the beam waist position along the laser beam path in the BNNT sample may then affect the SH signal intensity depending on the thickness of the sample. It is clear that for thicker samples, a lens with a longer focal length is preferred for evaluating the thickness uniformity. However, using a longer focal length will increase the focal spot size of the excitation beam and thus reduce the spatial resolution of the SH measurements. There is, therefore, a trade-off between how accurately the uniformity of the sample thickness can be measured and the spatial resolution of these measurements.

**Figure 6:**
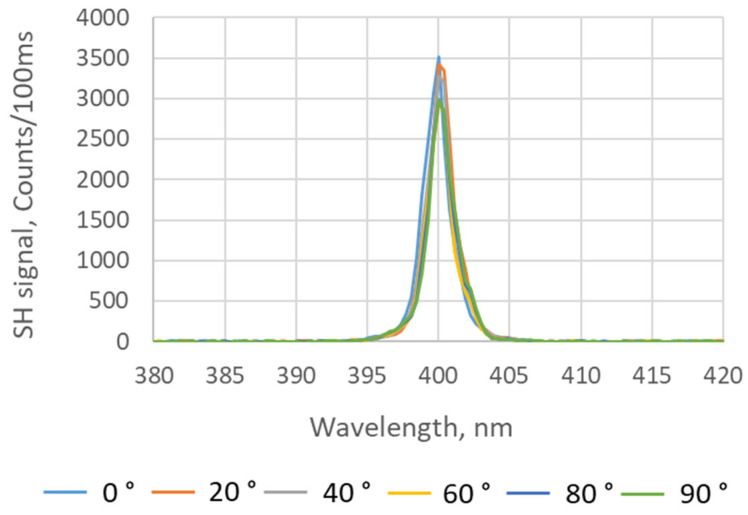
SH response of the 35 µm thick BNNT sheet for different orientations of the linear polarization of the excitation light with respect to the horizontal plane.

The SH response of an individual BNNT has strong dependence on the polarization of the excitation light. The experimental results showed that the maximum SH response of an individual multiwalled BNNT with a chiral angle of zero occurs when the polarization of the excitation light is parallel to the BNNT’s axis [34]. In the BNNT sheets, BNNTs and BNNT bundles are randomly orientated on a large scale, as shown in Figure 4c. However, on a scale of a few microns, which matches the focal spot size of the incident fs-IR laser beam, we can still observe a certain degree of BNNT alignment and, as a result, the SH signal may have some dependence on the polarization of the excitation light. In order to verify this experimentally, different polarization states of the excitation light were produced by rotating the half-wave plate (Figure 1), and the resultant SH responses of the BNNT sheets were measured. Figure 6 shows the polarization-dependent SH response of the 35 µm thick BNNT sheet for measurements performed in one location. The 18 and 60 µm thick BNNT sheets were tested as well and showed similar behavior. The angle indicated in Figure 6 refers to the angle between the linear polarization of the excitation light and the horizontal plane. The variations in the SH response caused by such changes in the polarization orientation were within ±8%, which can be deduced from Figure 6. The dependence of the SHG on the polarization orientation was also measured at different locations on the BNNT sheet; the maximum variation in the SH signal could be up to ±27% when the polarization of the excitation light was varied. The variations in the SH signal depended on the SH signal strength and were larger for stronger SH signals.

### 2.3. SHG in BNNT Coatings on Silica Substrates

Alternatively, BNNTs were deposited onto silica substrates with two coating approaches: the drop-casting process and the dip-coating process. In the drop-casting process, a drop of BNNT water solution was placed onto a silica substrate, and after the water completely evaporated at room temperature, another drop of the BNNT solution was placed at the same location and allowed to dry out in the air. By repeating the above steps, the thickness of the BNNT coating could be controlled. As for the dip-coating process, it was successfully developed in our previous studies for optical fiber sensors [35,36] and, hence, justifiably applied in this work to thin-square silica substrates. The silica substrates were dipped into a BNNT solution for 30 min, then slowly (0.2 mm/s) drawn out of the BNNT solution with a stepper motor and allowed to dry out in the air for 30 min. The above steps were repeated until a desired coating thickness was obtained.

**Figure 7:**
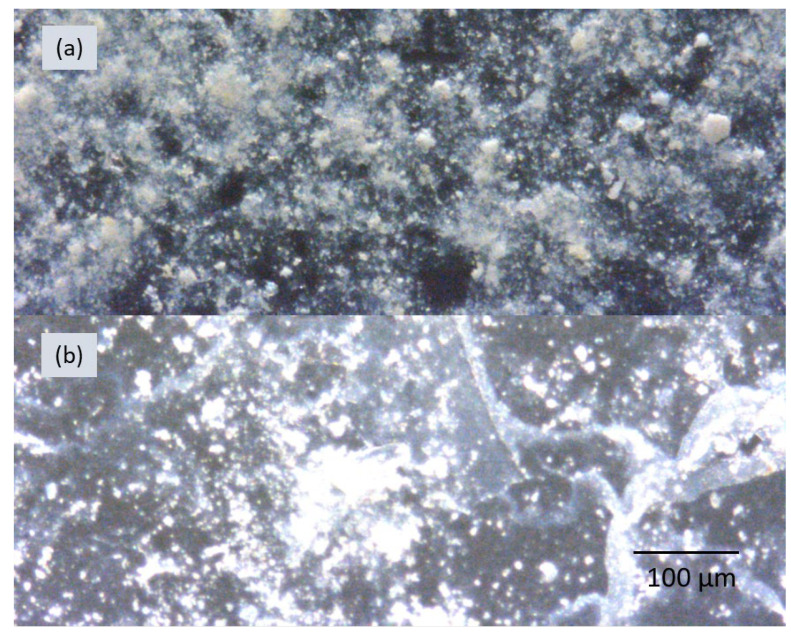
Optical microscopy images of BNNT coatings on silica substrates produced using the drop casting process (**a**) and dip-coating process (**b**).

In order to create a good affinity of BNNTs to the glass substrate and achieve at least partial alignment of BNNTs on the substrate, the top half of the silica substrate was passivated with 48% hydrofluoric acid (HF) for 2 min before initiating the dip-coating process. The HF etching of the top part of the silica substrate surface facilitated adherence and binding of the BNNTs to it. The bottom part of the silica substrate was not subjected to HF passivation and had a lower affinity to the BNNTs. The BNNTs were then deposited on the top part of the substrate first and, then, were self-stretched in the vertical direction when the substrate was pulled out of the solution. As a result, partial alignment of the BNNTs could be achieved. Figure 7 shows some representative optical microscopy images of BNNT coatings on silica substrates produced using the two coating processes. The BNNT coating shown in Figure 7a was produced with 10 drops of BNNT solution. The BNNT coating shown in Figure 7b was produced with 274 dip-coating cycles.

The coating thickness was measured by taking optical microscopy images of the silica substrate’s edge. For both coating processes, the thickness varied from 0 to 10 µm depending on the location, which is visualized in Figure 7. As one can see, the coatings are non-uniform, with the formation of BNNT clusters clearly observed. This phenomenon is inherent to the BNNT coating process, during which the solvent (i.e., water) takes a long time to evaporate and the suspended BNNTs quickly agglomerate at different locations on the substrate during the solvent evaporation. A thicker coating produced by applying more coating cycles may have an improved uniformity and give a more consistent SH response. However, a compromise between the coating thickness and the strength of the SH signal should also be found. Indeed, whereas a thicker coating may have smaller thickness variations, it could also weaken SHG because scattering of the SH signal on the increased number of different interfaces and boundaries is expected to be stronger. Finding a recipe to produce an optimum coating in the above context is the subject of our ongoing work.

**Figure 8:**
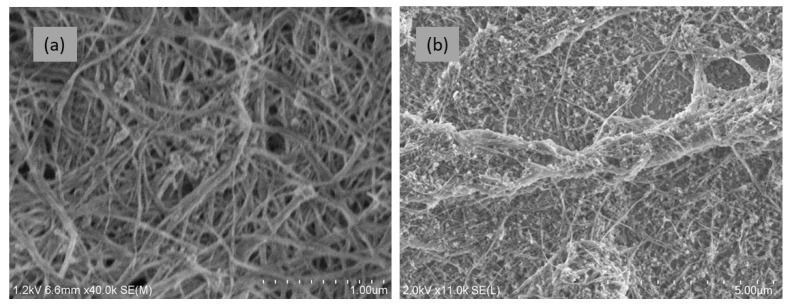
SEM images of BNNT coatings on silica substrates produced using the drop-casting process (**a**) and dip-coating process (**b**).

In order to evaluate the differences in the two coating processes, only thin BNNT coatings on silica substrates were examined. In this case, the analysis was performed using SEM to achieve the necessary high spatial resolution (Figure 8). The SEM image in Figure 8a shows that individual BNNTs and BNNT bundles in the coatings produced by drop-casting deposition are well delineated and randomly orientated on the surface, similar to the BNNT sheets (Figure 4c). However, when the dip-coating process was used, large and long BNNT bundles made of many BNNTs were produced on the silica surface (Figure 8b). The BNNTs in the bundles were partially aligned, which led to a higher SH response and stronger dependence on the polarization of the excitation laser beam.

**Figure 9:**
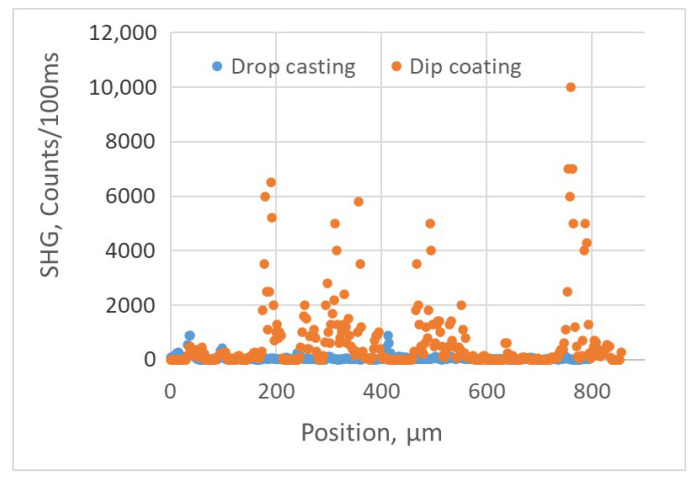
SH response of BNNT coatings produced by using the drop-casting and dip-coating processes at different locations on the samples.

Similar to the BNNT sheets, the SH responses of the BNNT coatings on silica substrates varied significantly depending on the locations on the surface. The spatial variations of the SH responses of the two types of coatings were measured at locations 2.5 μm apart when the focused excitation fs-IR laser beam was scanned across the samples (Figure 9), with the incident laser power being 160 µW at a frequency of 1 KHz. One can see from Figure 9 that the SHG in the BNNT coating made using the dip-coating method is much stronger than in the BNNT coating made using the drop-casting method, with the average SH responses being, respectively, 634 and 52 counts/100 ms. This indicates that alignment of BNNTs in the assemblies produced by dip coating is better than in the assemblies produced by drop casting, even though this is not clearly seen in the SEM images of Figure 8. As shown in Figure 7, the BNNT coatings on silica substrates were not uniform and, occasionally, there was an absence of BNNT coating over some regions of the substrate, resulting in a lack of SH response (see Figure 9). The experimental results shown in Figure 9 also confirm the conclusion from the earlier theoretical studies that well-aligned BNNT assemblies would produce strong SH responses [33]. Hence, increasing the alignment of BNNTs in the assemblies to achieve strong SHG is an important part of our ongoing efforts.

**Figure 10:**
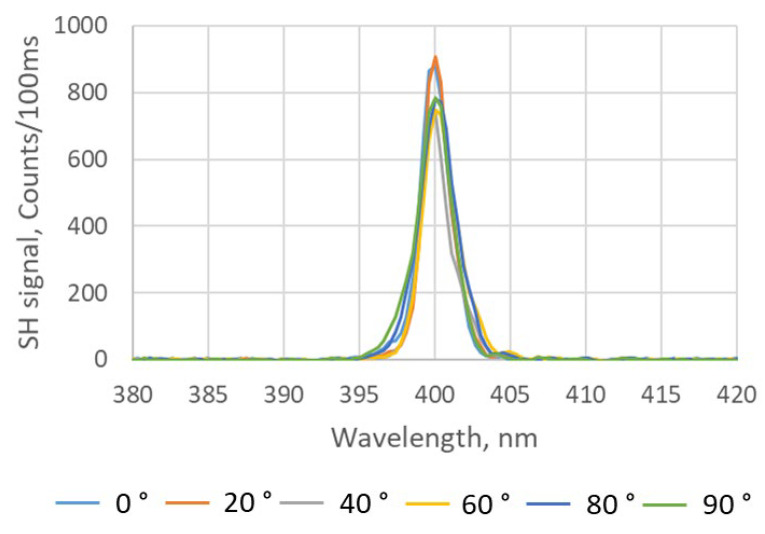
Polarization-dependent SH response of a BNNT coating produced by using the drop-casting process. Different orientations of the linear polarization of the excitation light with respect to the horizontal plane are indicated in the legend.

**Figure 11:**
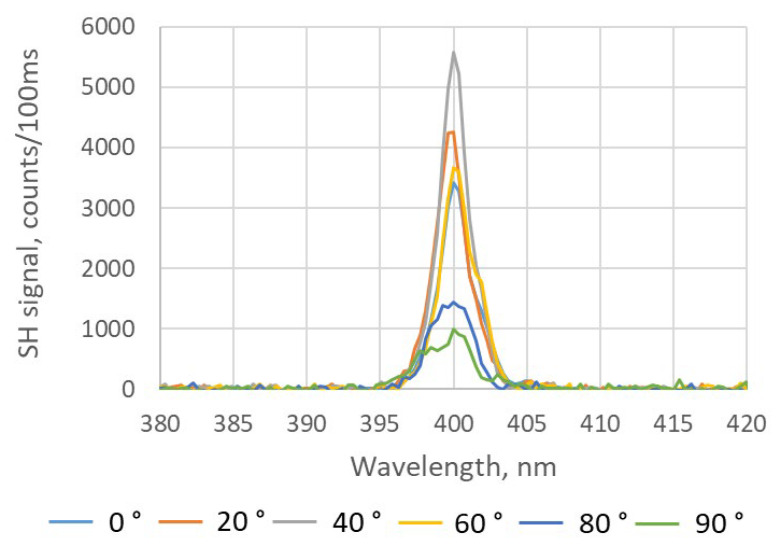
Polarization-dependent SH response of a BNNT coating produced by using the dip-coating process. Different orientations of the linear polarization of the excitation light with respect to the horizontal plane are indicated in the legend.

The polarization dependence of the SH responses of different BNNT assemblies is shown in Figure 10 and Figure 11, respectively. In the case of the drop-casting process, the average SH response is 807 counts/100 ms with a variation range of ±9% for different polarization states of the fs-IR laser, which is similar to the cases of the free-standing BNNT sheets. For the dip-coating process, the count rate varied by 72% from 1000 to 5500 counts/100 ms, which is much larger than that of any other BNNT assemblies measured in this work. Strong SHG exhibiting a pronounced dependence on the polarization state of the incident excitation light indicates that there is more alignment in the BNNTs and BNNT bundles, which is consistent with the SEM images shown in Figure 8b. It was shown experimentally that the intensity of the SH signal from an individual BNNT had a strong dependence on the polarization of the excitation light [34]. However, when a BNNT assembly is composed of randomly orientated BNNTs, there is expected to be no pronounced polarization dependence of the SH signal. Additionally, the BNNTs in an assembly are not identical as they can have different chirality, diameter, length, number of walls, etc. As a result, the polarization dependence of the SH response of individual BNNTs is unlikely to be the same as the SH response of BNNT assemblies which often have only weak polarization dependences. This is the case for BNNT sheets produced by a vacuum filtration method and BNNT coatings produced by a drop-casting process. However, when the BNNTs are partially aligned in a BNNT assembly, such as in a BNNT coating produced by a dip-coating process, there exists a preferred polarization state that produces a strong (or weak) SH signal from the assembly. This represents an averaging of the polarization dependency of each individual BNNT investigated by the excitation laser beam. Other parameters, such as sample thickness and material density, may also contribute to the enhancement of the SH response of the BNNT coating produced with the dip-coating process, but the BNNT alignment is the dominant factor, as evidenced by the strong dependence of the SH response on the polarization of the excitation laser beam.

The data to produce graphs in Figure 11 were collected at a location with a strong SH signal. At locations with a weaker SH signal, the polarization dependence of the SH signal was weaker. This is likely due to the reduced alignment of the BNNTs and BNNT bundles at those locations. The results shown in Figure 9 and Figure 11 indicate that for applications requiring a strong SH response, the dip-coating process is preferred provided that the polarization state of the excitation light is adjusted properly.

## 3. Conclusions

In this study, the SH responses of BNNT macroscopic assemblies were successfully measured for the first time. The experimental results showed that the SH responses vary significantly when the focus of the excitation fs-IR laser beam is moved along the BNNT macroscopic assembly, implying that the thickness of the local BNNT microstructure and alignment of BNNTs within it are variable. Thus, our SHG-based diagnostics technique can be used to assess the uniformity and surface roughness of BNNT macroscopic assemblies. The strong dependence of SH responses of the BNNT macroscopic assemblies on the degree of nanotube alignment can also be employed to optimize the production process based on the magnitude of the generated SH signal. If a certain application requires a strong SH response from the BNNT assemblies, the dip-coating process is preferred as compared to the drop-casting process, provided that the polarization of the incident excitation light is tuned accordingly to maximize the SH signal. The experimental results presented in this work can be applied for future research on the characterization of nanomaterials and their nonlinear applications such as wavelength conversion, encrypted communication, and optical sensors.

## Data Availability

The datasets generated and/or analyzed during the current study are available from the corresponding author upon reasonable request.

## Abbreviations

The following abbreviations are used in this manuscript:

BNNT	Boron nitride nanotube
SHG	Second harmonic generation
SH	Second harmonic
Fs-IR	Femtosecond infrared
h-BN	Hexagonal boron nitride
CNT	Carbon nanotube
